# Using machine learning to guide targeted and locally-tailored empiric antibiotic prescribing in a children's hospital in Cambodia

**DOI:** 10.12688/wellcomeopenres.14847.1

**Published:** 2018-10-10

**Authors:** Mathupanee Oonsivilai, Yin Mo, Nantasit Luangasanatip, Yoel Lubell, Thyl Miliya, Pisey Tan, Lorn Loeuk, Paul Turner, Ben S. Cooper

**Affiliations:** 1 Mahidol-Oxford Tropical Medicine Research Unit, Faculty of Tropical Medicine, Mahidol University, Bangkok, Thailand; 2 Division of Infectious Disease, University Medicine Cluster, National University Hospital, Singapore, Singapore; 3 Cambodia-Oxford Medical Research Unit, Angkor Hospital for Children, Siem Reap, Cambodia; 4 Center for Tropical Medicine and Global Health, Nuffield Department of Medicine, University of Oxford, Oxford, UK

**Keywords:** resistance, antimicrobial, antibiotic, machine learning, prediction, neonate

## Abstract

**Background**: Early and appropriate empiric antibiotic treatment of patients suspected of having sepsis is associated with reduced mortality. The increasing prevalence of antimicrobial resistance reduces the efficacy of empiric therapy guidelines derived from population data. This problem is particularly severe for children in developing country settings. We hypothesized that by applying machine learning approaches to readily collect patient data, it would be possible to obtain individualized predictions for targeted empiric antibiotic choices.

**Methods and Findings**: We analysed blood culture data collected from a 100-bed children's hospital in North-West Cambodia between February 2013 and January 2016. Clinical, demographic and living condition information was captured with 35 independent variables. Using these variables, we used a suite of machine learning algorithms to predict Gram stains and whether bacterial pathogens could be treated with common empiric antibiotic regimens: i) ampicillin and gentamicin; ii) ceftriaxone; iii) none of the above. 243 patients with bloodstream infections were available for analysis. We found that the random forest method had the best predictive performance overall as assessed by the area under the receiver operating characteristic curve (AUC). The random forest method gave an AUC of 0.80 (95%CI 0.66-0.94) for predicting susceptibility to ceftriaxone, 0.74 (0.59-0.89) for susceptibility to ampicillin and gentamicin, 0.85 (0.70-1.00) for susceptibility to neither, and 0.71 (0.57-0.86) for Gram stain result. Most important variables for predicting susceptibility were time from admission to blood culture, patient age, hospital versus community-acquired infection, and age-adjusted weight score.

**Conclusions**: Applying machine learning algorithms to patient data that are readily available even in resource-limited hospital settings can provide highly informative predictions on antibiotic susceptibilities to guide appropriate empiric antibiotic therapy. When used as a decision support tool, such approaches have the potential to improve targeting of empiric therapy, patient outcomes and reduce the burden of antimicrobial resistance.

## Introduction

There is consistent evidence that early and appropriate treatment of sepsis can reduce mortality ^1^. Since the definitive identification of a bacterial pathogen and its antibiotic susceptibility typically take three to four days using conventional culture methods, empiric antibiotic therapy (i.e. therapy that starts before the causative organism and its antibiotic susceptibility is known) is recommended. Choice of empirical antibiotic aims to balance two objectives: first, to cast a wide spectrum of coverage effective against the most likely causative organisms; second, to minimize the selection of resistance to reserve antibiotics for the wider population ^2^. Balancing the consequences associated with these two concerns - immediate patient outcomes and long-term resistance patterns impacting on future patients - represents a major challenge.

Empiric antibiotic choice for invasive bacterial infections in hospitalized children in low-to-middle income countries (LMICs) constitutes a particularly stark example of this problem owing to the high attributable mortality ^3^, and the high prevalence of antimicrobial resistance, particularly in neonates ^4^.

Current World Health Organization (WHO) guidelines for suspected sepsis or serious bacterial infection in newborns recommend empirical usage of gentamicin and ampicillin as the first line therapy, and change to third-generation cephalosporins if there is a lack of improvement in 24–72 hours ^5, 6^. However, a systematic review of community-acquired neonatal sepsis in developing countries in 2012 found that of the causative pathogens in older infants (1–12 months), only 63% and 64% showed *in vitro* susceptibility to ampicillin and gentamicin, and third-generation cephalosporins, respectively ^6^. For neonates, susceptibilities were even lower, with only 57% and 56% of pathogens susceptible to ampicillin and gentamicin and third-generation cephalosporins, respectively.

The potential harms of widespread antimicrobial resistance in children were illustrated in a recent study performed between 2007 and 2016 in a Cambodian children’s hospital, which found those infected with third-generation cephalosporin-resistant bacteria were less likely than others to receive appropriate antimicrobial therapy (57% vs. 94%), and when appropriate therapy was administered, it was initiated later ^7^. While anticipated clinical efficacy is the primary deciding factor in empirical antibiotic choices ^8^, there are other important considerations as well. These include side effect profile ^9^, cost, ease of administration and risks of promoting resistance emergence in hospital settings ^2^.

The adoption of antimicrobial stewardship programmes in hospitals is widely advocated internationally. This is true both in LMICs and high income countries ^10, 11^. Locally-adapted hospital antibiotic policies are important components of such programmes, and typically contain recommendations for empiric antibiotic use. In most cases, these recommendations are derived from expert opinion and informal (non-quantitative) syntheses of available evidence ^12^. In some cases simple decision support systems based on logistic regression models and scoring systems have been developed to help identify patients at high risk of being infected with multidrug-resistant pathogens. These approaches have primarily been developed in high- and upper middle-income countries ^13– 18^. The use of predictive modelling as part of clinical decision support systems for antimicrobial management remains rudimentary, with only one example identified in a recent systematic review ^19^. It has, however, been demonstrated in a randomized trial (in Israel, Germany and Italy) that a computerized decision support system making use of an underlying causal probabilistic network model can lead to more appropriate empiric antibiotic prescribing ^20^.

We hypothesized that applying modern machine learning approaches to readily collected patient data can surpass the performance of those based on logistic regression or simple decision trees, and derive patient-specific predictions for antibiotic susceptibility. Improved predictions directing empirical antibiotic therapy may contribute to better patient outcomes while avoiding the overuse of in-appropriate antibiotics that select for resistance.

In this study, we propose a locally adapted decision support system for a Cambodian children’s hospital by applying an array of machine learning algorithms to patient-level data. We evaluated the ability of the algorithms to predict whether the causative organisms are susceptible to: i) ampicillin and gentamicin; ii) ceftriaxone; iii) none of the above. We specifically focus on the value of using the predictive models to identify patients at high risk of being infected with organisms resistant to ceftriaxone, a third-generation cephalosporin, the most commonly prescribed empirical antibiotic in practice at our study site.

## Methods

### Data collection

Retrospective data were collected from the Angkor Hospital for Children, a non-governmental hospital in Siem Reap, Northwestern Cambodia with approximately 100 beds, and its Satellite Clinic situated 30km away, with 20 inpatient beds. The hospital provides free surgical and general medical care to children less than 16 years of age and is equipped with an intensive care unit (ICU). Admitted neonates and children come from both urban and rural settings, with about two thirds residing in Siem Reap province. Over 90% of inpatients come from the community, and the rest are transferred from another hospital. None of the children are born in the hospital as there is no obstetric service.

Blood cultures are routinely taken from febrile inpatients (axillary temperature *>* 37.5°C) in accordance with clinical algorithms. Processing of these cultures including *in vitro* antibiotic susceptibility testing has been described elsewhere ^21^. Children with at least one positive blood culture between February 2013 and January 2016 were included in the present study. Bloodstream infections with organisms that are likely skin contaminants such as coagulase-negative *Staphylococci*, Gram-positive bacilli, and mixed growths of environmental Gram-negative bacilli were excluded. We collected routine clinical and living conditions data, including household size, presence of domestic animals, and factors relating to water and sanitation.

The study was approved by the Angkor Hospital for Children Institutional Review Board (AHC-IRB, 290) and the Oxford Tropical Research Ethics Committee (OxTREC, 08-12). Written consent for the use of the patient data was obtained from the guardians of the children.

### Data analysis

We evaluated a suite of machine learning algorithms based on their ability to predict the invasive pathogens’ Gram stain and *in vitro* susceptibility to antibiotics using available information prior to receiving culture results. Specifically, we considered susceptibility to: i) ampicillin and gentamicin; ii) ceftriaxone; iii) none of the above. In the event that more than one organism was grown from the same blood culture, they were categorized as susceptible to the specified antibiotics only if all organisms were susceptible to at least one.

To predict the above antibiotic susceptibilities, we selected 35 independent variables (predictors) from patient records by coding quality and relevance. Dichotomous predictors where all but ten or fewer patients had the same value were excluded. Missing data for binary predictors were treated as negative.

Weight for age standard deviations (z-score), a measure of malnutrition, was calculated using the lambda, mu, and sigma (LMS) method ^22^ based on growth charts from the Centers for Disease Control. An earlier version of this article is available on BioRxiv as a preprint https://doi.org/10.1101/367037.

### Training the algorithms

We first performed a logistic regression with backwards step-wise AIC model selection ^23^. Additional machine learning algorithms were then explored, including decision trees constructed via recursive partitioning ^24^, random forests ^25^, boosted decision trees using adaptive boosting ^26^, linear support vector machines (SVM) ^27^, polynomial SVMs, radial SVMs ^28^ and *k*-nearest neighbours ^29^. All analysis was done in R version 3.5.1 ^30^ using the following packages: MASS ^31^ (stepwise logistic regression); rpart ^32^ (decision tress); ranger ^33^ (random forest); fastAdaboost ^34^ (boosted decision trees); kknn ^35^ ( *k*-nearest neighbors); kernlab ^36^ (polynomial and radial SVM); and LiblineaR ^37^ (linear SVM and regularized logistic regression).

Machine learning models were five-fold cross-validated. Data were randomly partitioned into five parts, with one part randomly held out for error estimation. An average of three repeats was taken to calculate the error for parameter fitting. Parameters were fitted for highest Kappa based on a grid search ^38^.

The data set was split 80/20 for training and testing purposes. For categorical variables we ensured that each category is represented by at least one record in the training set. To assess performance in predicting antibiotic susceptibility patterns, each model was refitted to 1,000 random selections of training and testing data sets. Performance was compared based on area under the receiver operating characteristic curve (AuROC) from the test set. We select the best method overall, then consider its probability calibration and the most important predictors. Variable importance in random forests was calculated using the method described in *Janitza et al.* ^39^.

### Identifying the optimum cut-off

The ROC curve describes the diagnostic ability of a binary classifier system, and plots the true positive rate (or sensitivity, i.e. the chance of correctly identifying a non-susceptible infection) against the false positive rate (or 1-specificity, i.e. the chance of incorrectly concluding an infection is non-susceptible). From this it would be possible to derive an optimal cutoff to maximize the overall test *accuracy* (i.e. the chance the test gives a true positive or true negative results). However, choosing the cut-off in this way would fail to account for the different health and economic costs of the two types of misclassification error (predicting resistance to an antibiotic when an organism is susceptible, and predicting susceptibility when an organism is resistant). A more rational approach is to choose the test cutoff to maximize overall utility, taking into account the different numbers of expected false positives and false negatives associated with different cutoffs and the different health-economic impacts of these two misclassification errors. These include costs of antibiotic prescriptions, excess length of stay, mortality as a result of inappropriate empiric antibiotic prescriptions and, most challengingly, future impact of resistance selection resulting from different antibiotic prescribing decisions. Because the cost of future resistance is difficult to quantify, we adopt an alternative approach by considering willingness to pay (WTP) for avoiding unnecessary use of carbapenems (where such use is considered unnecessary if the organism is susceptible to a first line antibiotic). With this economic framework, and using conventional recommendations for WTP per quality adjusted life year (QALY) gained ^40^, health impact and monetary costs can be combined on the same scale and represented as net monetary value (monetary loss + QALY loss *×* WTP). In this way, we can assign different net monetary values to each of the four possible test outcomes (true positive, true negative, false positive, false negative). The optimal cutoff for utility will be a value of the specificity that minimizes this net monetary loss. We provide illustrative examples of these calculations (see S3 Appendix for further details) and provide a user-friendly web application to enable optimal cutoffs to be determined under different assumptions, available at http://moru.shinyapps.io/ahc-ml-amr-cost/.

## Results

**Figure 1.**
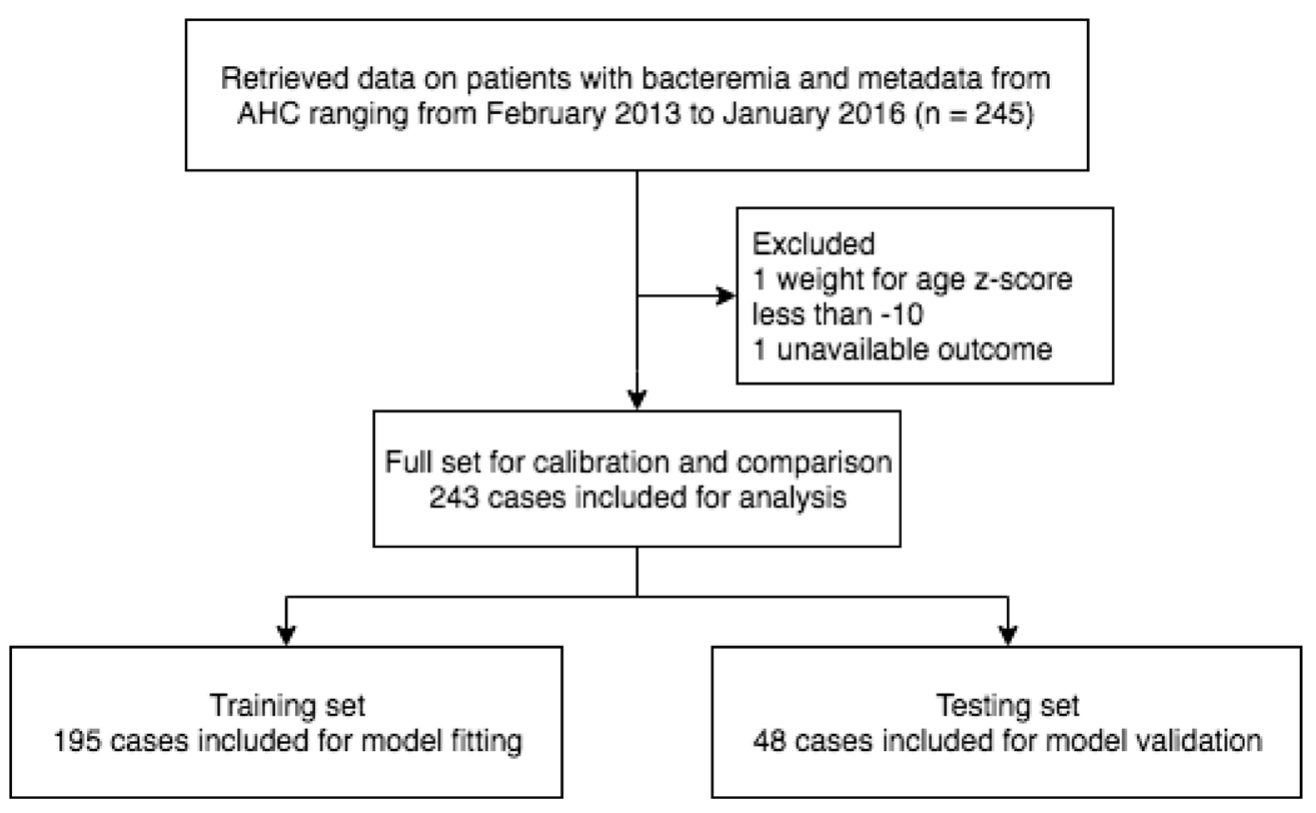
Selection of records.

Figure 1 shows the selection of cases used for model training and testing. Of 245 cases, two cases were excluded; one due to missing target outcome data, and the other due to a biologically impossible value.

**Figure 2.**
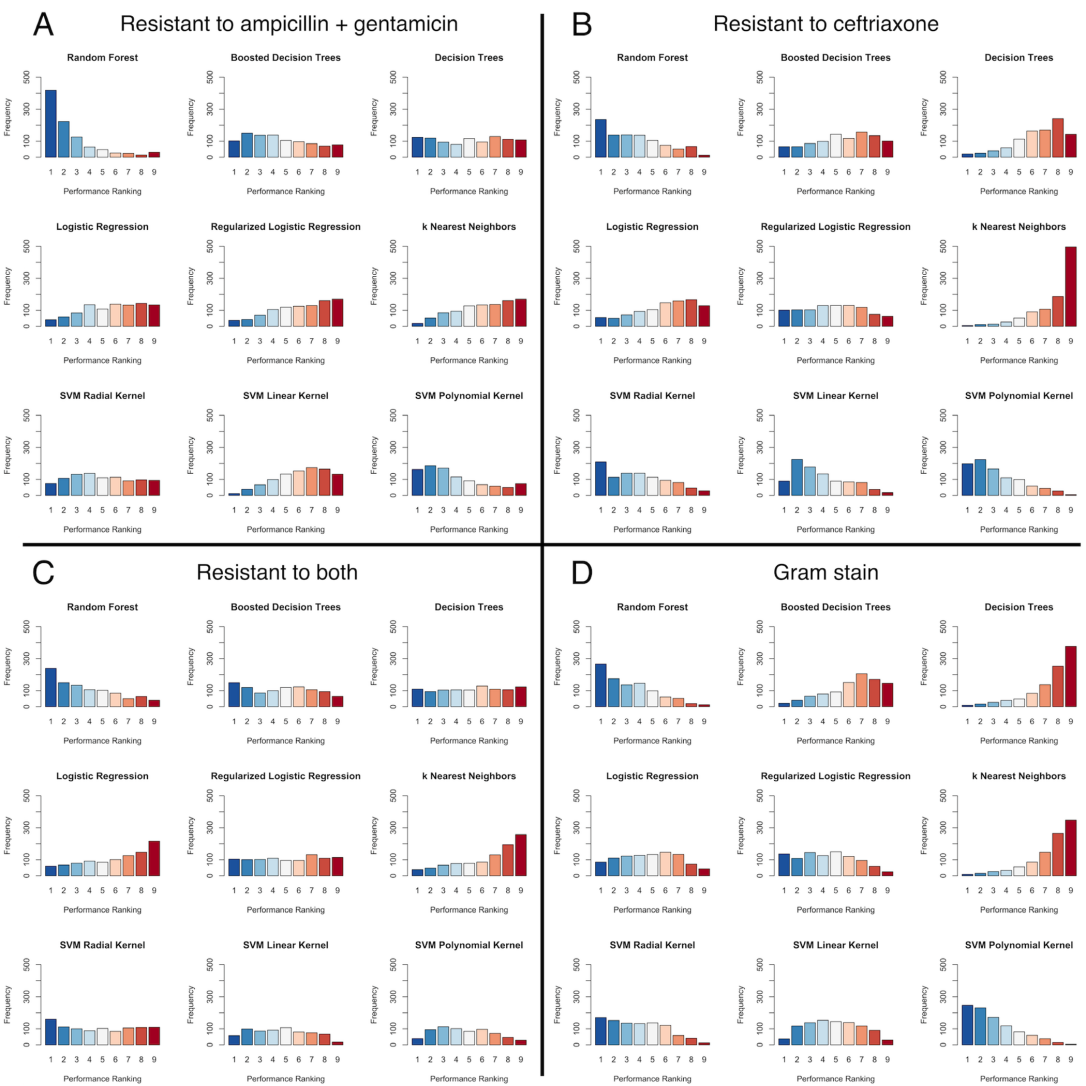
Comparison of performance rankings. Histograms of performance rankings obtained with 1000 random splits of the data into training (80%) and testing (20%) sets for the eight machine learning algorithms for predicting four outcomes ( **A**) Resistance to ampicillin and gentamicin ( **B**) Resistance to ceftriaxone ( **C**) Resistance to ampicillin and gentamicin, and ceftriaxone ( **D**) Gram stain. A ranking of 1 (blue) is best, 9 (red) is worst, based on the area under the receiver operating characteristic curve (AuROC) with the test data.

Based on the AuROC derived from the test data set, the random forest method is the most frequently ranked first ( Figure 2A and 2C), and was consistently superior to decision trees, boosted decision trees, *k*-nearest neighbours, and the widely-used stepwise logistic regression. The performance of SVM approaches is generally good, but varies with the kernel the models were based on and which outcomes were being considered. For example, an SVM with polynomial kernel has similar performance to the random forest approach when predicting resistance to ceftriaxone ( Figure 2B), but performed poorly in predicting lack of susceptibility to all three of the antibiotics ( Figure 2C).

**Figure 3.**
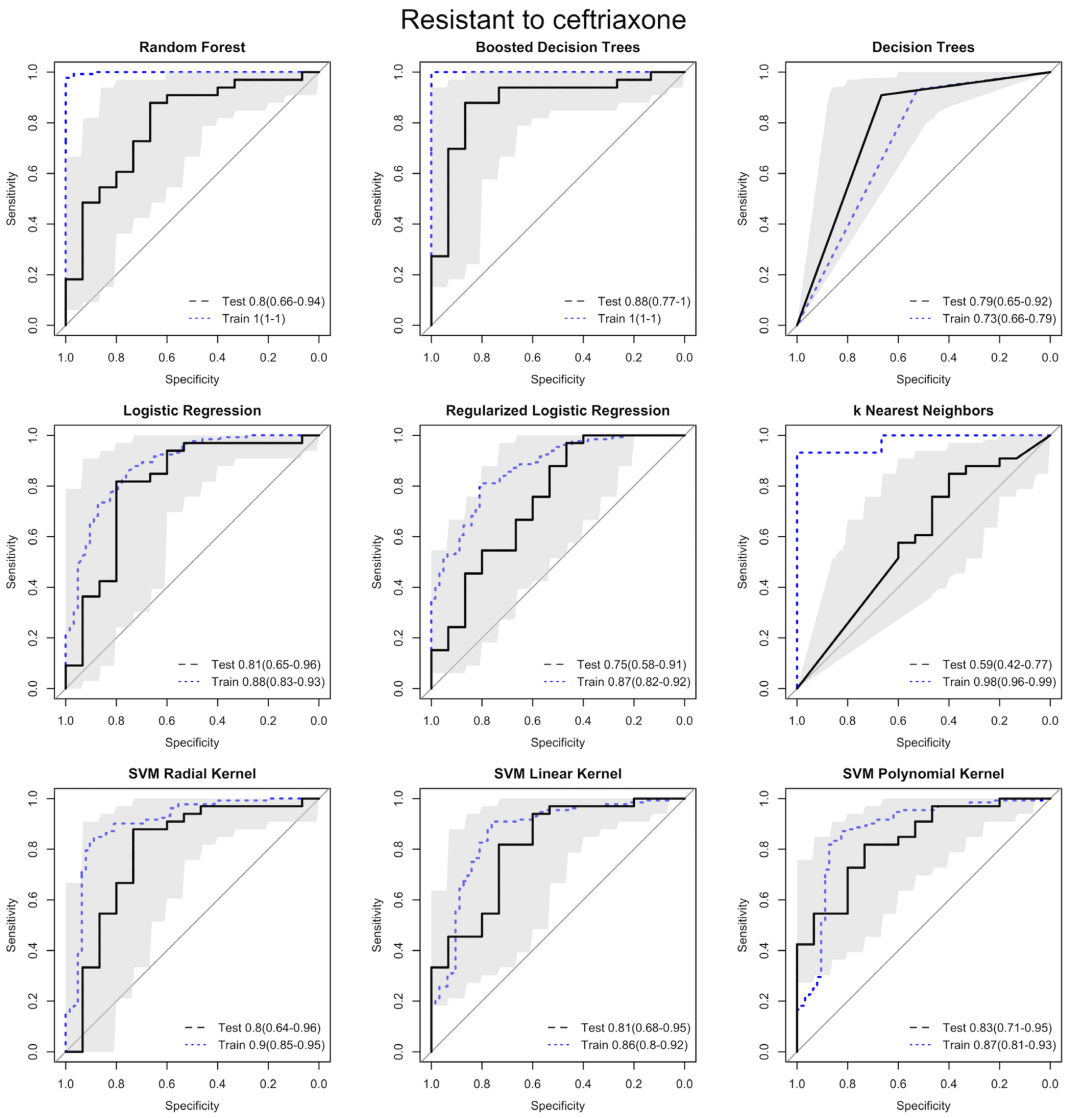
Receiver Operating Characteristic (ROC) curves for predicting resistance to ceftriaxone. Training set (blue dotted line), testing set, i.e. predictive performance (black solid line with 95% confidence intervals shown by shading). The diagonal line represents the line of no-discrimination, or the expected performance of a random guess.

Ranking, although a good indicator of relative performance, does not necessarily indicate prediction ability itself. A comparison between multiple low AuROCs could still give a top ranked winner despite having low AuROC values. Figure 3 shows the Receiver Operating Characteristic (ROC) curves for predicting lack of susceptibility to ceftriaxone for all methods. This figure highlights the disconnect between predictive performance on the training data set (blue dotted line) and that on the test set (black dashed lines), highlighting the importance of separating the training and testing data. ROC curves show tradeoffs between specificity and sensitivity. If ROC curves for different methods were plotted on the same plot, it is possible for ROC curves for different methods to cross, indicating that optimal methods may vary depending on the cutoff used, and that the methods with the highest AUC may not always be the best for a given application. Importantly, the random forest test set ROC curve did not cross with other test set ROC curves. S1 Appendix shows ROC curves for remaining outcomes.

**Figure 4.**
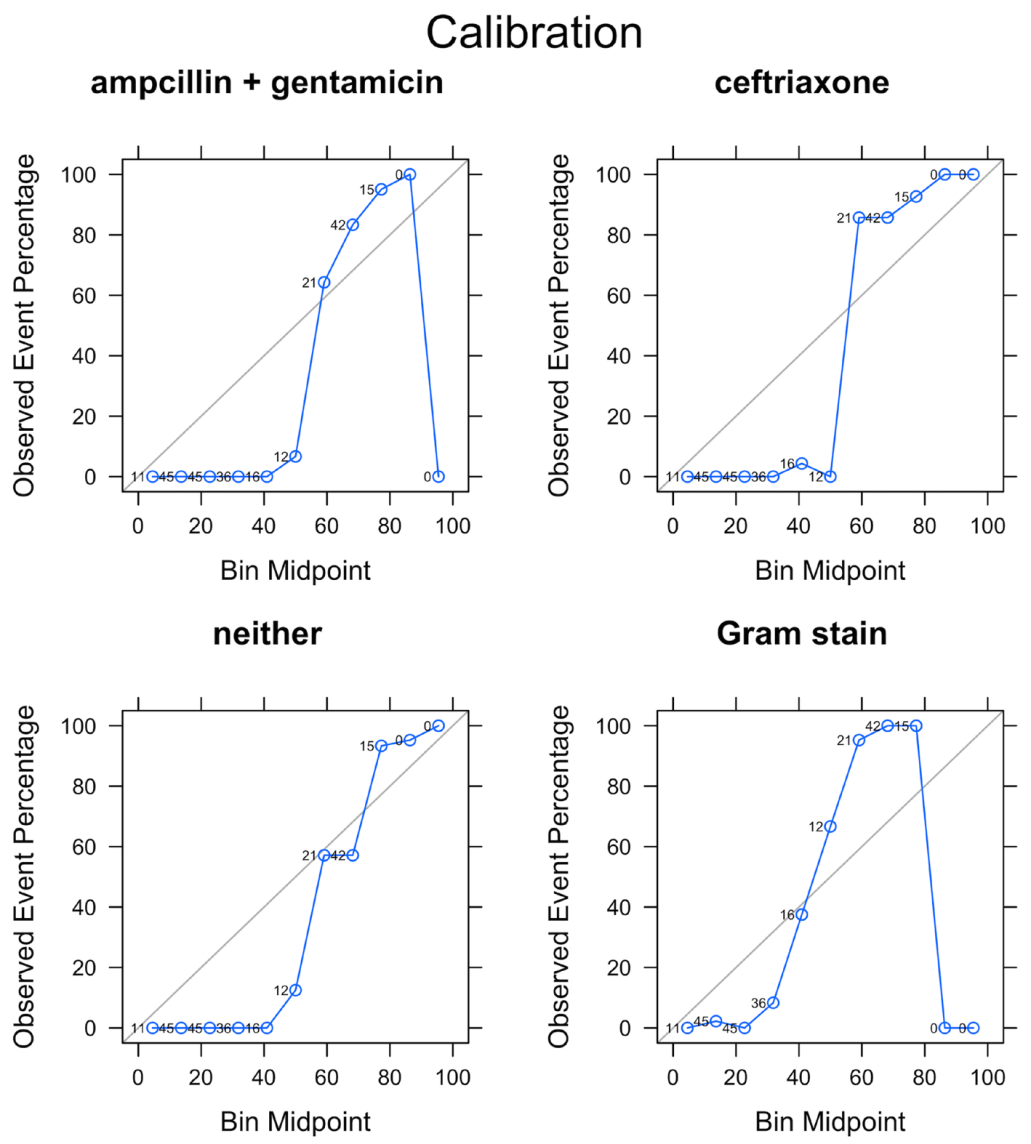
Calibration plots for random forest algorithm. This compares predicted probabilities (grouped into 10 equal-sized bins on x-axis) to observed event frequencies in real data (y-axis) using the entire data set. Points close to the grey diagonal line indicate that the predicted probability is close to the observed frequency. Numbers above points indicate the number of records contributing to each point.

To be effective in supporting decisions, it is useful to not only rank well (predict correctly), but also to be well-calibrated (i.e. the estimated probabilities that pathogens lack susceptibility to an antibiotic should be similar to observed frequencies). Calibration refers to coherence between these estimated probabilities and the observed frequencies. To illustrate this, a calibration plot for the prediction of resistance to ceftriaxone with the random forests algorithm is shown in Figure 4. This shows that even though the random forests method gives high accuracy (i.e. has a high AuROC), in this particular case it tends to be overconfident in its prediction probability. This overconfidence in prediction could not be improved even after adjustment with isotonic regression or Platt scaling ^41^.

**Figure 5.**
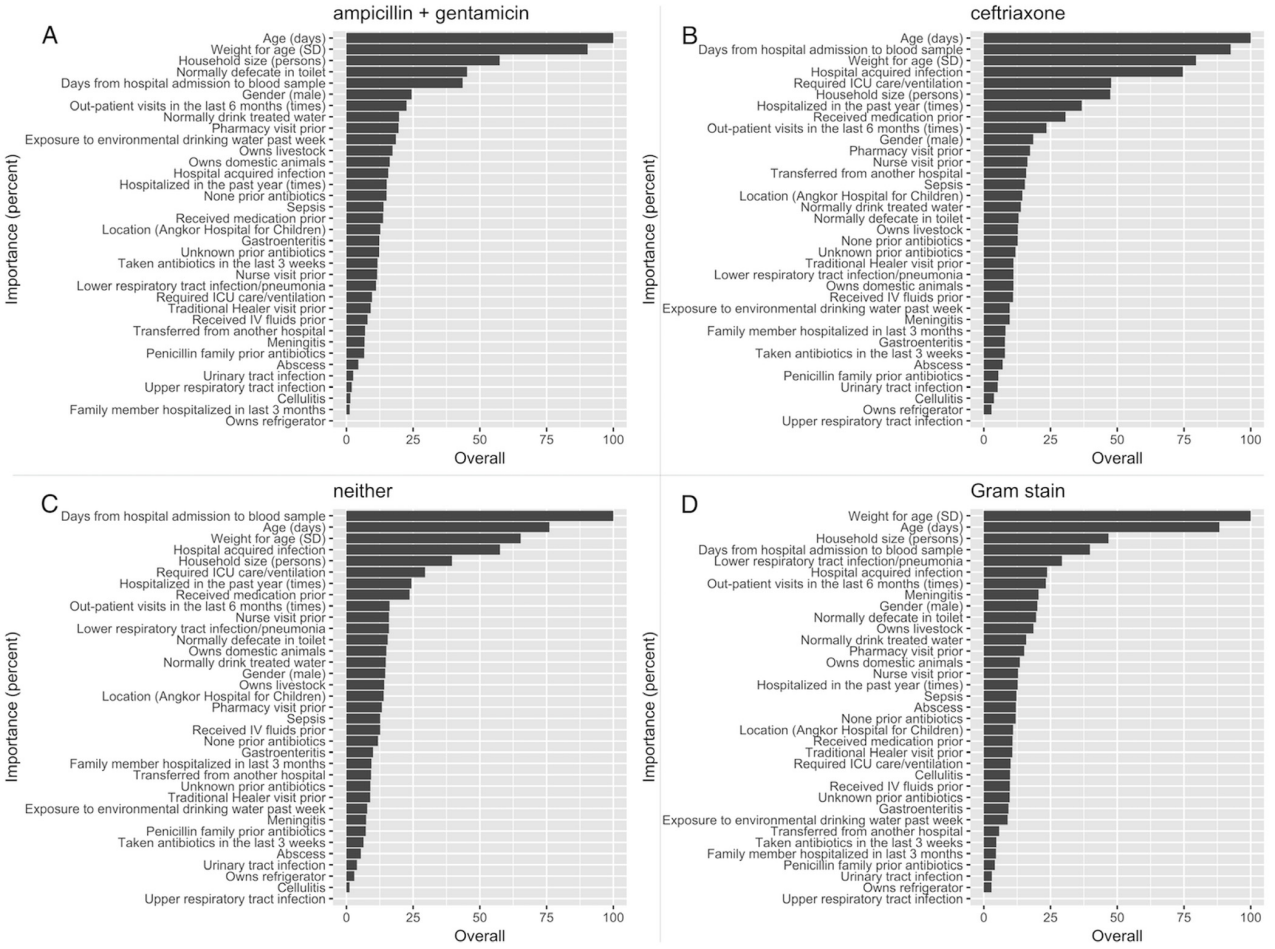
Importance of predictors in random forest models. Results show the relative importance of variables for predicting resistance to ampicillin + gentamicin ( **A**) resistance to ceftriaxone ( **B**) resistance to all three antibiotics ( **C**) Gram stain ( **D**).

Figure 5 illustrates the influence of each independent variable on the random forest model in predicting antibiotic susceptibilities ^39, 42^.

This shows that the most important predictor for resistance to ceftriaxone is patient age (leaving this out would decrease the model accuracy 100% of the time). Patient age is closely followed by days from hospital admission to blood sample, age-adjusted weight score, and the classification of the infection as hospital- or community-acquired (omitting this variable would decrease model accuracy 75% of the time). Other variables had much smaller effects.

The most important predictors in the random forest model for the other three outcomes were broadly similar. Interestingly, the classification of infection as hospital- or community-acquired had less importance for predicting resistance to ampicillin and gentamicin compared to ceftriaxone, but household size was found to much more important.

**Figure 6.**
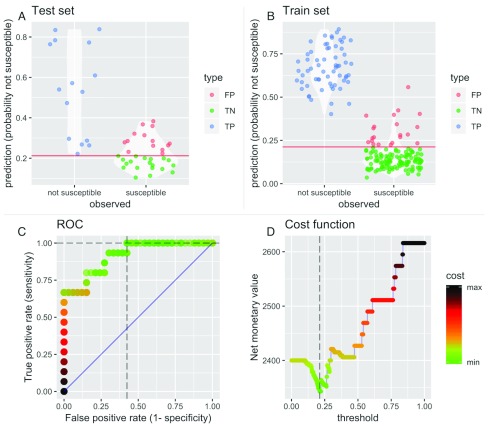
Effects of test cutoff on decision outcomes and utility. Panels **A** and **B**: Impact of test threshold (horizontal red line in panels **A** and **B**) on classification of resistance to ceftriaxone into false negatives (FN), false positives (FP), true negatives (TN) and true positives (TP) in test (panel **A**) and training (panel **B**) data. Panel **C**: The ROC curve is shaded according to utility loss at different cutoffs, where horizontal dashed lines correspond to the threshold selected by minimizing the cost function ( **D**), i.e. maximising utility. Higher utilities, i.e. lower costs (expressed as a net monetary value) are shaded in green. Interactive version available at http://moru.shinyapps.io/ahc-ml-amr-cost/

Figure 6 illustrates how, used as part of a decision support system, the choice of test threshold to inform antibiotic prescribing decisions would impact on the number of patients treated empirically with appropriate antibiotics. Taking a test threshold of 0.21 for the predicted probability that ceftriaxone would not be an effective treatment (so above this value, patients would be recommended to receive a second-line antibiotic, typically a carbapenem, instead of ceftriaxone), 15 out of 15 (100%) patients in the test data set who have ceftriaxone-resistant infections would be correctly identified (true positives). This threshold choice would also lead to 14 of the 33 (42%) patients with ceftriaxone-susceptible infections unnecessarily receiving the second-line antibiotic (over-treatment). Adjusting the threshold corresponds to moving the red line in Figure 6A–B up and down, changing the numbers of patients over- and under-treated. The choice of this threshold has an impact on patient outcomes and costs; their combined impact can be represented as the net utility loss (expressed as a net monetary value) due to infection ( Figure 6D). A rational approach would be to choose the threshold to minimize this utility loss. However, quantifying utility loss due to future selection for resistance when using antibiotics is challenging ^43^, so an alternative approach is to choose a prediction threshold based on clinical judgment, and work backwards to determine how this implicitly values the utility loss due to over-treatment. In this example, we find a threshold of 0.21 implies that we would be willing to pay $US 200 to avoid one unnecessary course of a carbapenem. Details of the calculations can be found in the supplementary text ( S3 Appendix).

## Discussion

Our results show that modern machine learning algorithms can outperform widely-used logistic regression models and provide predictions about antibiotic susceptibility that could potentially be used to improve empirical antibiotic prescribing. We found that the random forest approach performed particularly well, especially for predicting ceftriaxone resistance, the most widely used empiric antibiotic for our study patients. To our knowledge this is the first time such machine learning algorithms have been applied to this problem in a hospital setting.

The most important variables for predicting antibiotic susceptibility were found to be time from admission to blood culture, patient age, age-adjusted weight score, and hospital versus community-acquired infection. These are objective and routinely collected variables available in most clinical settings. All other variables included in the models are also easily collected at minimal cost through short questionnaires. The computations underlying the predictions can readily be performed in a few seconds on a low-cost computer, or remotely via any device connected to the Internet. This makes the approach highly suitable for other LMIC settings, which typically face the highest disease burden and the most urgent problems with antimicrobial resistance ^44^. These machine-learning models, which are often assumed to depend on large datasets more commonly available in high-income settings ^45^, may be of considerable value even in resource-limited and relatively data-poor settings.

### Wider implications

Used as part of a decision support system, the best machine learning approaches should, in theory, make it possible to substantially increase the proportion of patients who receive effective empiric antibiotics, while minimizing the risks of increased resistance selection that would be associated with a blanket change in the default choice of empiric antibiotics for all patients. Clearly, further work is needed to evaluate such deployment in practice ^46^.

Rapid microbiological diagnostic tests offer an alternative pathway for improving the precision of early antibiotic prescribing. Affordable and accurate tests are not currently available, but this situation may change in the coming years. While machine learning approaches proposed here could be considered a stopgap, we think it is more likely that the two approaches will be complementary. Results from future rapid diagnostic tests could be used as inputs in machine learning algorithms along with other patient variables, and would be expected to lead to more reliable predictions than those from the rapid tests alone.

### Utility

A common dilemma in designing diagnostic systems is to identify the optimal cutoff point for sensitivity and specificity on the ROC curve. Increasing the sensitivity threshold for detecting antibiotic resistance will capture more cases of resistance, but will inadvertently lead to more false positives, resulting in increased prescriptions of unnecessary broad-spectrum antibiotics and selection for resistance. Conversely, while setting the threshold at higher specificity will reduce false positives, the model will miss more patients with resistant bacterial infections, leading to delayed prescription of appropriate antibiotics. A natural approach would be to choose the cutoff to maximise utility (which includes health outcomes and opportunity costs associated with economic costs). While quantifying the direct health care cost components is relatively straightforward, the costs of resistance are far more challenging to calculate. Shrestha *et al.* estimated the costs of resistance per antibiotic consumed, assigning a cost of $US 0.8 and $US 1.5 per standard unit of carbapenem in Thai and US settings, respectively ^43^. However, these estimates did not take into account the potentially grave potential consequence of losing a ’last-line’ antibiotic to resistance. Better quantification of the cost of resistance is an important area of future research ^47^.

### Strengths and limitations

We systematically evaluated a number of machine learning algorithms to determine the algorithms with the best predictive performance. Most currently available clinical scoring systems rely on logistic regression models, probably for historical reasons. No method is universally better than another method ^48, 49^, however different algorithms have strengths and weaknesses and our results suggest that by focusing on a single learning algorithm, much of the previous literature may have missed an important opportunity.

It is possible that a more extensive exploration of logistic regression models would have yielded better results (for example by including interaction terms and variable transformations). However, such complexities are rarely considered in practice and would impose a substantially greater burden on the analyst than the simple "cookbook" approaches considered in this study.

A second important strength of our work is that algorithm training and evaluation were performed on different data sets. Though there are some notable exceptions ^13, 15, 18^, this separation has not always been done in previous attempts to predict antibiotic susceptibility. As is clearly shown in Figure 3, if this separation is not done true predictive power is likely to be substantially lower than reported.

Thirdly, our analysis uses single-hospital data for formulating and evaluating models. If we had used a large dataset aggregated from multiple settings in the hope of increasing generalisability, the algorithm performance would have likely suffered. Scoring systems developed in one setting have been found to have substantially worse performance in different settings ^14, 50^. By applying many different models to the same data set, our approach focuses on generalizing predictions toward new events within the same setting ^51^.

There are several limitations to our study. The trade-off with a setting-specific predictive system is the likely poor predictive value when applied in another setting ^14, 50^. Wider deployment of such approaches would require models to be tailored to local data. The model may also become less relevant as time passes. Identifying the most appropriate temporal and spatial selection windows for training data is an important area for future research.

### Understanding the algorithms

One potential obstacle to the wider adoption of machine learning algorithms is that, to many, they are a black box. An intuitive way to understand them is to consider a geometric interpretation. Suppose we have a dataset with two predictors, height and weight and one binary outcome, diseased or healthy. We then plot a graph with weights on the x-axis and height on the y-axis. We can imagine each data point inhabiting a point in this 2-dimensional graph plane, *feature space*. Each point would have a label of the class we are trying to predict (i.e. diseased/healthy). A classification problem can be likened to a search to find a line (or lines) which best separates the data points with different labels on its feature space. In this example, this refers to a line which splits between the diseased and healthy on the height-weight graph. For two independent variables this plane is 2 dimensional. For *n* predictors this would require *n*-dimensions. For *n >* 3 this is harder to visualize, but the geometric interpretation still holds.

A geometric visualization allows us to appreciate the varying performances of each method by considering how each method arrives at the conclusion as to which line (or combination of lines) is best. A decision tree can be considered a combination of decisions, each represented by a line in our feature plane (i.e. *is weight > 50 kg?* can be considered a line at 50 on the weight axis). A combination of simple lines allows for more complex decision boundaries. However, because of their ability to create complex boundaries, they tend to over-fit. Random forests are designed to correct for the over-fitting by decision trees by building a consensus of a multitude of decision trees, and averaging these trees by giving the majority vote after polling all component decision trees based on classification.

**Table 1.** Distribution of variables for logistic regression for susceptibility to ceftriaxone.

Characteristics	Treatable n = 127 (No/Yes)	Resistant n = 68 (No/Yes)	OR (univariate)	95% CI	P-value
Age (days)	703; 1063 *	1616; 1613 *	1.00	1.00-1.00	<0.001
Complication during admission					
Required ICU care/ventilation	32/31	23/109	0.20	0.10-0.40	<0.001
Transfer from another hospital	18/45	18/114	0.47	0.22-1.02	0.057
Admission differential diagnosis					
Sepsis	37/26	82/50	1.19	0.64-2.21	0.581
Meningitis	4/59	23/109	2.63	0.86-8.06	0.090
Lower respiratory tract infection/pneumonia	17/46	33/99	0.83	0.42-1.65	0.596
Upper respiratory tract infection	3/60	6/126	1.29	0.33-5.04	0.714
Gastroenteritis	9/54	18/114	0.95	0.40-2.25	0.902
Cellulitis	4/59	11/121	0.95	0.28-3.29	0.937
Abscess	2/61	10/122	1.08	0.32-3.65	0.902
Urinary tract infection	2/61	12/120	7.36	0.95-57.25	0.057
Weight for age (SD)	-2.2; 1.7 *	-2.1; 1.7 *	1.00	0.84-1.20	0.968
Hospitalised in the last year (times)	0; 0-3 ‡	0; 0-3 ‡	0.50	0.30-0.84	0.009
Out-patient visits in the last 6 months (times)	0; 0-3 ‡	0; 0-3 ‡	1.12	0.71-1.79	0.620
Treatment prior to current admission					
Pharmacy	8/55	43/89	3.80	1.67-8.66	0.001
Nurse	22/41	64/68	1.64	0.88-3.03	0.117
Traditional Healer (Khru Khmer)	8/55	15/117	0.77	0.32-1.87	0.562
Received IV fluids	11/52	31/101	1.23	0.59-2.55	0.576
Received medication	34/29	105/27	4.81	2.44-9.48	<0.001
Household size	6; 3-10 ‡	6; 3-10 ‡	1.06	0.93-1.20	0.403
Owns domestic animals	49/14	92/40	0.56	0.27-1.17	0.122
Owns livestock	44/19	89/43	0.79	0.42-1.49	0.463
Normally defecate in a toilet	33/30	62/70	0.83	0.45-1.51	0.537
Owns refrigerator	4/59	5/127	1.46	0.38-5.60	0.578
Taken antibiotics in the last 3 weeks	4/59	22/110	3.78	1.08-13.20	0.037
Family member hospitalized in last 3 months	5/58	9/123	0.85	0.27-2.65	0.777
Exposure to environmental drinking water in past week	7/56	24/108	2.15	0.88-5.24	0.091
Normally drink treated water	26/37	61/71	1.43	0.78-2.63	0.249
Hospital acquired infection	34/29	11/121	0.07	0.03-0.16	<0.001
Days from hospital admission to blood sample	0; 0-104 ‡	0; 0-104 ‡	0.87	0.81-0.94	<0.001
Gender (Male)	33/30	79/53	1.27	0.70-2.33	0.430
Location (Angkor Hospital for Children)	54/9	94/38	0.28	0.12-0.66	0.004
Taken antibiotics prior to admission					
None (antibiotics)	39/24	62/70	0.51	0.28-0.95	0.033
Penicillin Family	4/59	17/115	2.32	0.84-6.45	0.106
Unknown	17/46	53/79	1.95	0.99-3.84	0.053

*Mean; SD for normal distributions, ‡Mode; Range for exponential distributions, SD, standard deviation; CI, confidence interval; OR, odds ratio; Inf, infinity Odds ratio from multivariate logistic regression analysis prior to step-wise backward elimination See S2 Appendix for other outcomes

## Conclusions

Decision support systems, informed by readily available setting-specific data, have the potential to lead to evidence-based hospital antibiotic policies which could improve appropriate prescribing of empiric antibiotics. This would be expected to lead to better patient outcomes and minimize the risk of antibiotic resistance emergence. While guidelines for developing a hospital antibiotic policy advocate conducting literature reviews and basing recommendations on local cumulative surveillance antibiograms ^12^, we have shown that machine learning algorithms informed by relatively small amounts of patient-level data can be used to derive patient specific predictions for empirical antibiotic therapy. Such a prediction system can be developed cheaply, using easily-collected data, and is well-suited to LMIC settings.

## Data availability

Zenodo: Manuscript dataset - Using machine learning to guide targeted and locally-tailored empiric antibiotic prescribing in a children’s hospital in Cambodia, http://doi.org/10.5281/zenodo.1256967 ^52^.

## Supplementary material

**S1 Appendix.** ROC comparisons for predicting for treatability by remaining outcomes: ampicillin + gentamicin, neither, and Gram stain. Blue dotted lines: training set, black dashed lines: testing test (actual performance).

**S2 Appendix.** Distribution of variables for logistic regression for treatability by remaining outcomes: ampicillin + gentamicin, neither, and Gram stain.

**S3 Appendix.** Economic model to identify the optimum cut off.
