# Supplementary material for: Using machine learning to guide targeted and locally-tailored empiric antibiotic prescribing in a children's hospital in Cambodia
Source: Wellcome Open Res. 2018 Oct 10;3:131. [Version 1] doi: 10.12688/wellcomeopenres.14847.1 (PMC6352926; doi:10.12688/wellcomeopenres.14847.1)
Supplement: Supplementary file 1 [file wellcomeopenres-3-16176-s0000.tgz › 32801df0-8828-4e1c-b6f2-1cd1772bcb13_Supplementary_File_1.pdf]

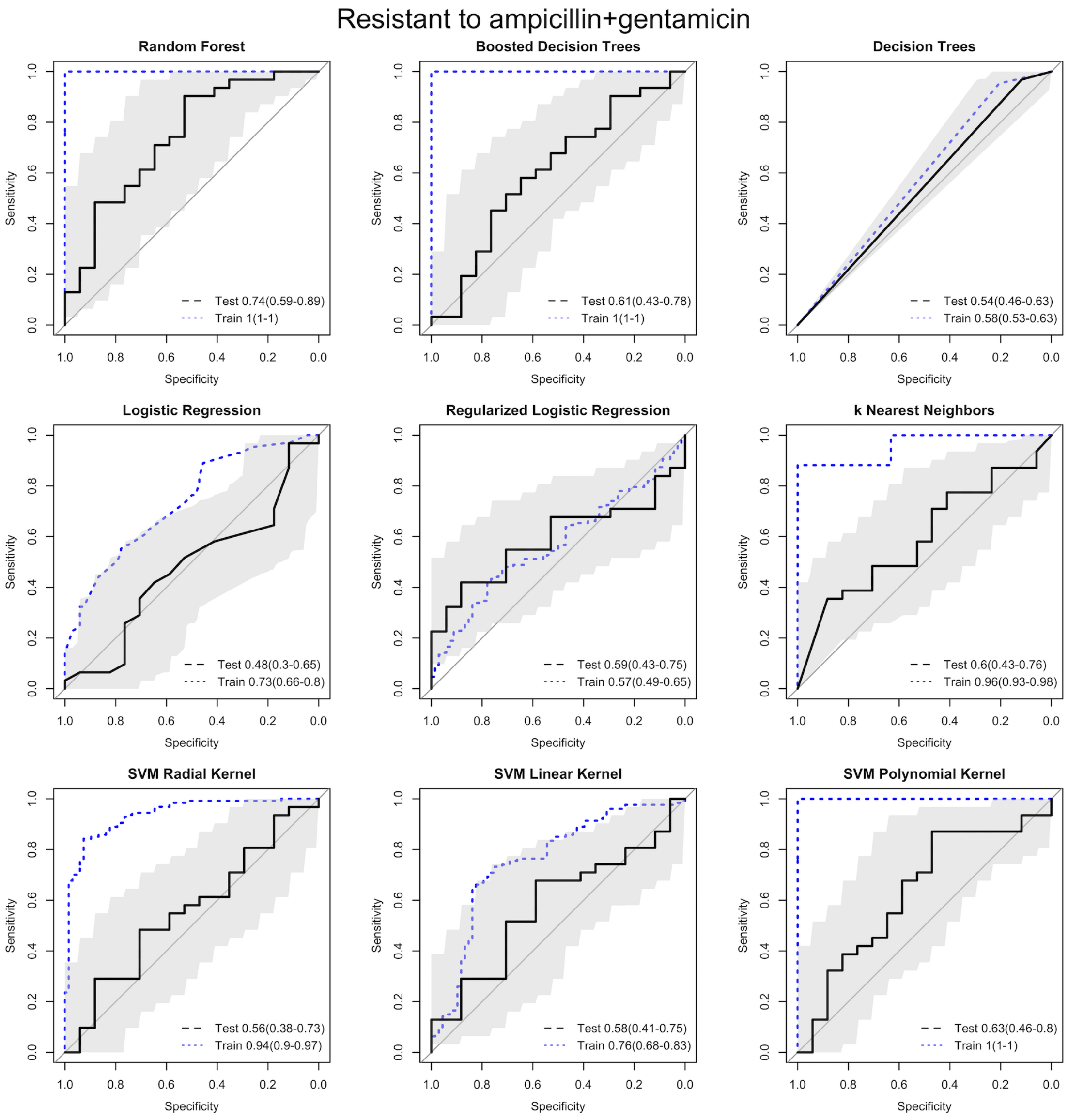

Figure 1: Receiver Operating Characteristic (ROC) curves for predicting lack of susceptibility to ampicillin + gentamicin. Training set (blue dotted lines), testing set i.e. actual performance (black dashed lines with 95% intervals shown by shading). The solid diagonal line is the line of no-discrimination, the expected performance of a random guess.

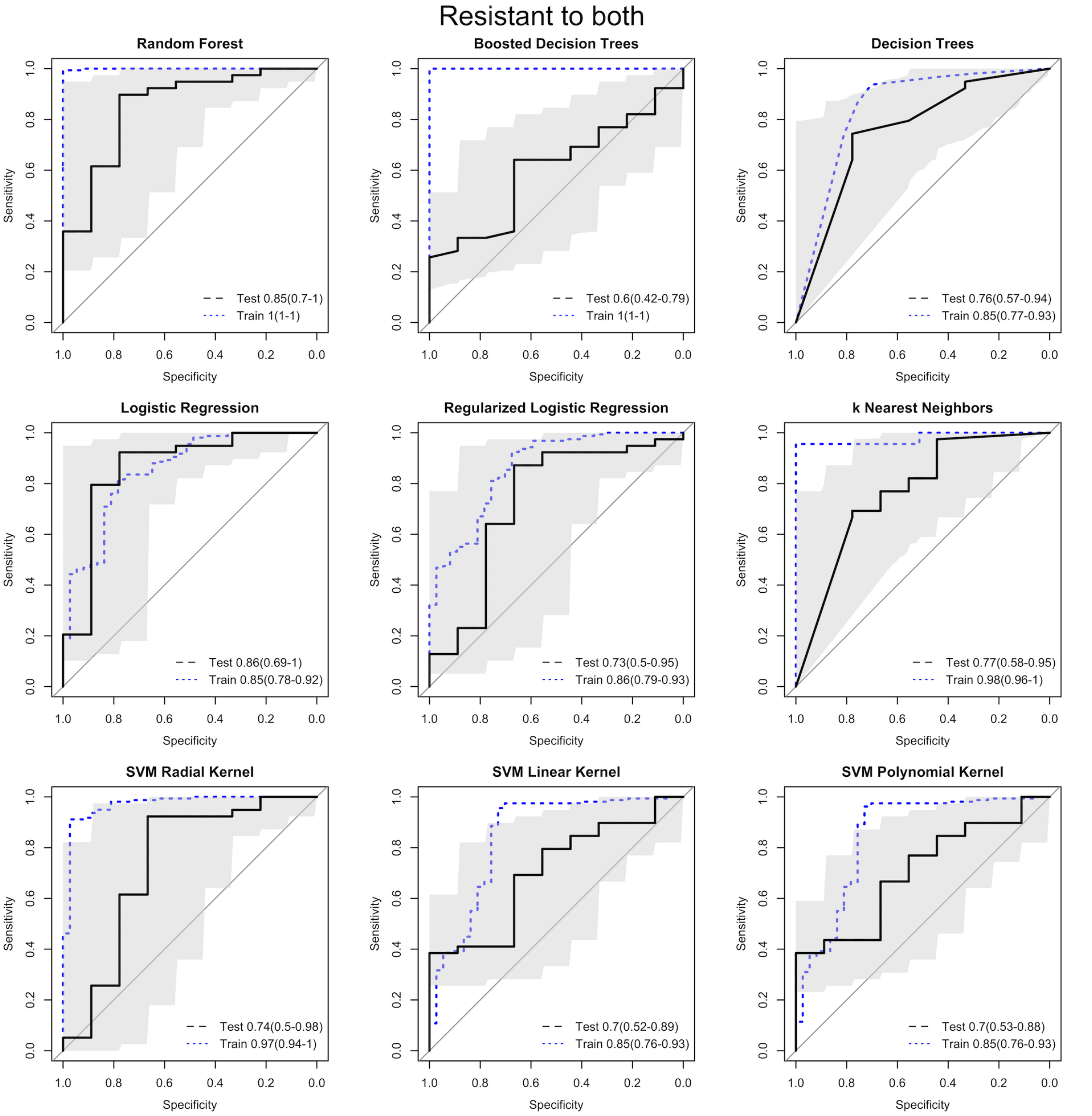

Figure 2: Receiver Operating Characteristic (ROC) curves for predicting lack of susceptibility to neither ampicillin + gentamicin nor ceftriaxone. Training set (blue dotted lines), testing set i.e. actual performance (black dashed lines with 95% intervals shown by shading). The solid diagonal line is the line of no-discrimination, the expected performance of a random guess.

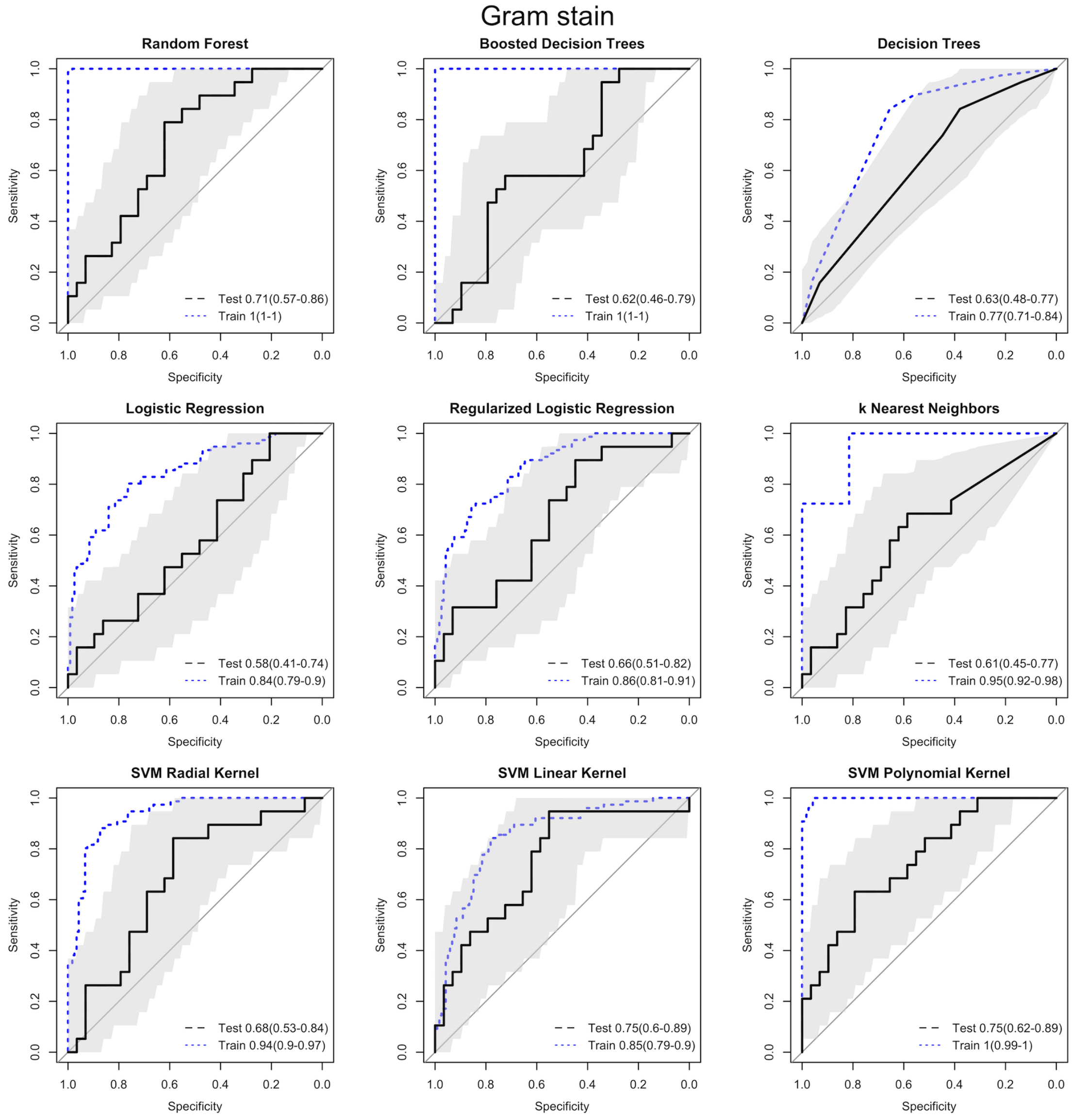

Figure 3: Receiver Operating Characteristic (ROC) curves for predicting Gram stain. Training set (blue dotted lines), testing set i.e. actual performance (black dashed lines with 95% intervals shown by shading). The solid diagonal line is the line of no-discrimination, the expected performance of a random guess.
