# Supplementary material for: Using machine learning to guide targeted and locally-tailored empiric antibiotic prescribing in a children's hospital in Cambodia
Source: Wellcome Open Res. 2018 Oct 10;3:131. [Version 1] doi: 10.12688/wellcomeopenres.14847.1 (PMC6352926; doi:10.12688/wellcomeopenres.14847.1)
Supplement: Supplementary file 2 [file wellcomeopenres-3-16176-s0001.tgz › be09eda8-d906-4e34-8894-9752be2fe49a_Supplementary_File_2.pdf]

Table 1: **Distribution of variables for logistic regression for susceptibility to ampicillin + gentamicin**

Characteristics	Treatable n = 127 (No/Yes)	Resistant n = 68 (No/Yes)	OR (univariate)	95% CI	P-value
Age (days)	1466; 1682*	1325; 1453*	1.00	1.00-1.00	0.366
Complication during admission					
Required ICU care/ventilation	21/42	30/102	0.52	0.28-0.98	0.045
Transfer from another hospital	14/49	22/110	0.87	0.40-1.91	0.733
Admission differential diagnosis					
Sepsis	40/23	78/54	0.98	0.54-1.79	0.951
Meningitis	5/58	20/112	3.17	1.04-9.65	0.042
Lower respiratory tract infection/pneumonia	16/47	32/100	0.98	0.50-1.90	0.941
Upper respiratory tract infection	3/60	5/127	1.07	0.26-4.44	0.921
Gastroenteritis	11/52	17/115	0.49	0.21-1.15	0.102
Cellulitis	3/60	9/123	6.99	0.89-54.97	0.065
Abscess	3/60	8/124	2.52	0.53-12.00	0.247
Urinary tract infection	5/58	11/121	1.67	0.52-5.39	0.391
Weight for age (SD)	-2.3; 1.5*	-2; 1.8*	1.20	1.00-1.44	0.057
Hospitalised in the last year (times)	0; 0-3‡	0; 0-3‡	0.96	0.61-1.50	0.851
Out-patient visits in the last 6 months (times)	0; 0-3‡	0; 0-3‡	1.29	0.79-2.10	0.313
Treatment prior to current admission					
Pharmacy	15/48	40/92	1.06	0.56-2.02	0.851
Nurse	26/37	58/74	0.75	0.42-1.37	0.353
Traditional Healer (Khru Khmer)	9/54	9/123	0.53	0.23-1.24	0.145
Received IV fluids	14/49	28/104	1.04	0.50-2.15	0.913
Received medication	41/22	100/32	1.96	1.03-3.74	0.040
Household size	5; 3-11‡	5; 3-11‡	1.02	0.90-1.14	0.794
Owns domestic animals	45/18	92/40	0.95	0.49-1.84	0.871
Owns livestock	42/21	82/50	0.99	0.54-1.82	0.973
Normally defecate in a toilet	29/34	64/68	2.11	1.16-3.86	0.015
Owns refrigerator	4/59	8/124	1.46	0.37-5.68	0.588
Taken antibiotics in the last 3 weeks	6/57	18/114	0.60	0.27-1.30	0.193
Family member hospitalized in last 3 months	6/57	9/123	1.08	0.31-3.71	0.908
Exposure to environmental drinking water in past week	16/47	16/116	0.47	0.22-1.01	0.053
Normally drink treated water	24/39	64/68	1.49	0.82-2.73	0.194
Hospital acquired infection	16/47	31/101	0.48	0.25-0.93	0.031
Days from hospital admission to blood sample	0; 0-48‡	0; 0-48‡	0.98	0.95-1.01	0.124
Gender (Male)	32/31	73/59	1.78	0.98-3.22	0.058
Location (Angkor Hospital for Children)	54/9	99/33	1.00	0.49-2.04	0.999
Taken antibiotics prior to admission					
None (antibiotics)	35/28	70/62	1.26	0.70-2.28	0.440
Penicillin Family	5/58	20/112	0.98	0.35-2.78	0.969
Unknown	20/43	42/90	0.86	0.47-1.58	0.632

*Mean; SD for normal distributions, ‡Mode; Range for exponential distributions,

SD, standard deviation; CI, confidence interval; OR, odds ratio; Inf, infinity

Odds ratio from multivariate logistic regression analysis prior to step-wise backward elimination

Table 2: **Distribution of variables for logistic regression for susceptibility to neither ampicillin + gentamicin nor ceftriaxone**

Characteristics	Treatable n = 127 (No/Yes)	Resistant n = 68 (No/Yes)	OR	95% CI	P-value
Age (days)	1185; 1415*	1591; 1589*	1.00	1.00-1.00	0.062
Complication during admission					
Required ICU care/ventilation	21/42	31/101	0.28	0.13-0.59	0.001
Transfer from another hospital	9/54	25/107	0.66	0.26-1.69	0.382
Admission differential diagnosis					
Sepsis	41/22	79/53	1.11	0.54-2.31	0.773
Meningitis	8/55	14/118	6.13	0.80-46.94	0.081
Lower respiratory tract infection/pneumonia	16/47	33/99	0.71	0.32-1.53	0.380
Upper respiratory tract infection	1/62	9/123	10564819.17	0.00-Inf	0.990
Gastroenteritis	10/53	14/118	1.48	0.48-4.55	0.496
Cellulitis	5/58	8/124	1.57	0.34-7.27	0.565
Abscess	3/60	7/125	0.93	0.25-3.48	0.916
Urinary tract infection	7/56	8/124	11085620.12	0.00-Inf	0.987
Weight for age (SD)	-2.1; 2.2*	-2.2; 1.5*	1.07	0.87-1.32	0.503
Hospitalised in the last year (times)	0; 0-2‡	0; 0-2‡	0.76	0.47-1.21	0.241
Out-patient visits in the last 6 months (times)	0; 0-2‡	0; 0-2‡	1.35	0.73-2.48	0.335
Treatment prior to current admission					
Pharmacy	23/40	41/91	1.98	0.82-4.82	0.131
Nurse	25/38	59/73	1.71	0.81-3.60	0.157
Traditional Healer (Khru Khmer)	10/53	14/118	0.52	0.20-1.36	0.180
Received IV fluids	8/55	33/99	0.99	0.42-2.37	0.989
Received medication	45/18	98/34	3.88	1.82-8.26	¡0.001
Household size	5; 3-15‡	5; 3-15‡	1.04	0.89-1.21	0.634
Owns domestic animals	49/14	92/40	1.00	0.46-2.19	0.998
Owns livestock	38/25	86/46	0.88	0.42-1.87	0.748
Normally defecate in a toilet	30/33	69/63	0.99	0.48-2.03	0.978
Owns refrigerator	6/57	7/125	2.69	0.34-21.53	0.350
Taken antibiotics in the last 3 weeks	8/55	20/112	6.45	0.84-49.26	0.072
Family member hospitalized in last 3 months	4/59	9/123	0.60	0.15-2.40	0.474
Exposure to environmental drinking water in past week	13/50	24/108	1.32	0.47-3.69	0.598
Normally drink treated water	28/35	62/70	1.79	0.84-3.81	0.132
Hospital acquired infection	17/46	26/106	0.11	0.05-0.25	¡0.001
Days from hospital admission to blood sample	0; 0-104‡	0; 0-104‡	0.96	0.93-0.99	0.019
Gender (Male)	27/36	75/57	1.64	0.80-3.37	0.178
Location (Angkor Hospital for Children)	54/9	98/34	0.17	0.04-0.76	0.020
Taken antibiotics prior to admission					
None (antibiotics)	33/30	68/64	0.82	0.40-1.69	0.594
Penicillin Family	7/56	16/116	1.55	0.43-5.54	0.501
Unknown	22/41	45/87	1.30	0.60-2.82	0.511

*Mean; SD for normal distributions, ‡Mode; Range for exponential distributions,

SD, standard deviation; CI, confidence interval; OR, odds ratio; Inf, infinity

Odds ratio from multivariate logistic regression analysis prior to step-wise backward elimination

Table 3: **Distribution of variables for logistic regression for Gram stain**

Characteristics	Treatable n = 127 (No/Yes)	Resistant n = 68 (No/Yes)	OR (univariate)	95% CI	P-value
Age (days)	1199; 1503*	1436; 1522*	1.00	1.00-1.00	0.873
Complication during admission					
Required ICU care/ventilation	23/40	34/98	0.81	0.42-1.57	0.531
Admission differential diagnosis					
	NA	NA	0.60	0.27-1.34	0.211
Transfer from another hospital	14/49	18/114	4.32	1.122-16.6	0.033330
Sepsis	36/27	91/41	1.85	1.01-3.40	0.047
Meningitis	6/57	18/114	5.02	1.87-13.50	0.001
Lower respiratory tract infection/pneumonia	20/43	36/96	2.88	1.52-5.45	0.001
Upper respiratory tract infection	1/62	6/126	0.77	0.19-3.19	0.723
Gastroenteritis	13/50	14/118	0.45	0.17-1.19	0.107
Cellulitis	1/62	10/122	3.92	0.98-15.67	0.053
Abscess	4/59	8/124	3.86	1.15-13.03	0.029
Urinary tract infection	5/58	11/121	0.86	0.28-2.67	0.795
Weight for age (SD)	-2.3; 1.8*	-2.1; 1.7*	1.22	1.02-1.47	0.034
Hospitalised in the last year (times)	0; 0-4‡	0; 0-4‡	0.64	0.37-1.11	0.110
Out-patient visits in the last 6 months (times)	0; 0-2‡	0; 0-2‡	1.02	0.67-1.55	0.926
Treatment prior to current admission					
Pharmacy	16/47	41/91	1.33	0.71-2.49	0.369
Nurse	25/38	59/73	0.92	0.52-1.65	0.789
Traditional Healer (Khru Khmer)	8/55	13/119	1.84	0.77-4.41	0.171
Received IV fluids	11/52	27/105	0.95	0.47-1.93	0.895
Received medication	41/22	98/34	1.14	0.60-2.15	0.695
Household size	6; 3-12‡	6; 3-12‡	0.92	0.81-1.04	0.191
Owns domestic animals	44/19	89/43	1.40	0.72-2.70	0.319
Owns livestock	37/26	81/51	1.20	0.65-2.19	0.561
Normally defecate in a toilet	27/36	70/62	1.85	1.03-3.31	0.040
Owns refrigerator	3/60	9/123	0.77	0.22-2.65	0.680
Taken antibiotics in the last 3 weeks	7/56	18/114	1.01	0.41-2.46	0.987
Family member hospitalized in last 3 months	5/58	8/124	0.40	0.11-1.50	0.175
Exposure to environmental drinking water in past week	11/52	22/110	0.78	0.36-1.68	0.531
Normally drink treated water	17/46	68/64	1.14	0.64-2.03	0.661
Hospital acquired infection	22/41	20/112	0.17	0.07-0.42	¡0.001
Days from hospital admission to blood sample	0; 0-48‡	0; 0-48‡	0.89	0.81-0.97	0.012
Gender (Male)	31/32	73/59	3.51	1.89-6.49	¡0.001
Location (Angkor Hospital for Children)	55/8	99/33	1.50	0.75-3.00	0.256
Taken antibiotics prior to admission					
None (antibiotics)	34/29	72/60	1.31	0.73-2.34	0.365
Penicillin Family	6/57	14/118	0.71	0.29-1.73	0.445
Unknown	22/41	44/88	0.91	0.49-1.69	0.768

*Mean; SD for normal distributions, ‡Mode; Range for exponential distributions,

SD, standard deviation; CI, confidence interval; OR, odds ratio; Inf, infinity

Odds ratio from multivariate logistic regression analysis prior to step-wise backward elimination
