# Supplementary material for: Using machine learning to guide targeted and locally-tailored empiric antibiotic prescribing in a children's hospital in Cambodia
Source: Wellcome Open Res. 2018 Oct 10;3:131. [Version 1] doi: 10.12688/wellcomeopenres.14847.1 (PMC6352926; doi:10.12688/wellcomeopenres.14847.1)
Supplement: Supplementary file 3 [file wellcomeopenres-3-16176-s0002.tgz › 80521104-17f2-4762-829b-623904ed9620_Supplementary_File_3.pdf]

S4 Appendix: Economic model to identify the optimum test threshold

To use prediction scores for lack of susceptibility to the default empiric antibiotic as part of a decision support system, a threshold score is needed above which a different antibiotic is recommended. A natural approach is to choose this threshold to maximize overall utility. Here we provide illustrative calculations to show how this can be done.

We focus on the decision problem of whether to use the default empiric antibiotic Ceftriaxone (a third-generation cephalosporin, to which a proportion of infections lack susceptibility), or the alternative, Imipenem (a carbapenem) to which we assume the prevalence of resistance is initially negligible. Use of Imipenem, however, has the additional "cost" of risking increasing future carbapenem-resistance, which would be expected to have a negative impact on future patient outcomes.

The approach we take is to first quantify the utility loss associated with a single patient under all four possible decision outcomes (True Positive, True Negative, False Positive, False Negative). As we adjust the decision threshold (which corresponds to raising or lowering the red line in Fig 6), the number of observations in the test data set that fall into each of the above four categories changes. For each threshold value we then simply sum up the utility loss associated with each case for a given threshold. The threshold giving the smallest utility loss is considered optimal.

To determine the utility loss associated with each outcome we need to account for: i) the difference in the risk of death for patients receiving effective and ineffective empiric antibiotics; ii) the difference in the length of stay and associated costs of patients receiving effective and ineffective empiric antibiotics; iii) quality adjusted life year (QALY) loss due to death; iv) willingness to pay per QALY gained; v) costs of the different antibiotics; and, most challengingly, vi) the expected additional future QALY loss of using Imipenem instead of Ceftriaxone as a result of selection of resistance to a last line antibiotic. All calculations are performed on a monetary scale, by multiplying QALY changes by willingness to pay for one QALY gain. Because vi) is particularly challenging to quantify, we instead express this as the willingness to pay to avoid prescribing Imipenem instead of Ceftriaxone. In the tables below, Table S1 summarizes the definitions of the four possible decision outcomes, Table S2 summarizes the calculations of the cost components, Table S3 does the same for associated health outcomes, and Table S4 provides parameter values and sources for the calculations used in our illustrative example.

Table S1: Descriptions

Population groups	Description
True Positive (TP)	A case with an infection that is not susceptible to Ceftriaxone where the algorithm correctly recommends using Imipenem
True Negative (TN)	A case with an infection that is susceptible to Ceftriaxone where the algorithm correctly recommends using Ceftriaxone
False Positive (FP)	A case with an infection that is susceptible to Ceftriaxone where the algorithm incorrectly recommends using Imipenem
False Negative (FN)	A case with an infection that is not susceptible to Ceftriaxone where the algorithm incorrectly recommends using Ceftriaxone

Table S2: Calculation of cost components for population groups

Population groups	Costs function
C_1 (TP)	Cost of Imipenem*Number of TP + WTP for avoiding unnecessary Imipenem use*Number of TP
C_2 (TN)	Cost of Ceftriaxone*Number of TN
C_3 (FP)	Cost of Imipenem*Number of FP + WTP for avoiding unnecessary Imipenem use*Number of FP
C_4 (FN)	Cost of Ceftriaxone*Number of FN + Cost of excess length of stay*Number of FN

Table S3: Calculation of health outcome components for population groups

Population groups	Utility function (in monetary terms)
U_1 (TP)	Risk of death from correct treatment*Number TP*QALYloss*WTP
U_2 (TN)	Risk of death from correct treatment*Number TN*QALYloss*WTP
U_3 (FP)	Risk of death from correct treatment*Number FP*QALYloss*WTP
U_4 (FN)	Risk of death from incorrect treatment*Number FN*QALYloss*WTP

Net Monetary Value

$$Net\ Monetary\ (NM) = \sum(C1 + C2 + C3 + C4) + \sum(U1 + U2 + U3 + U4)$$

Table S4: Parameters

Items	Values	Unit	Reference
Cost of gentamicin	0.13	US$ per day	DMSIC 2016
Cost of ampicillin	0.825	US$ per day	DMSIC 2016
Cost of ceftriaxone	0.45	US$ per day	DMSIC 2016
Cost of imipenem	7.5	US$ per day	DMSIC 2016
Number of empirical treatment (days)	3	days	Expert opinion
Excess length of hospital stay due to inappropriate empirical treatment	2	days	Marquet et al. 2015
Cost of hospital stay	7.65	US$ per bed day	WHO CHOICE
WTP for avoiding unnecessary Imipenem prescription	200	US$ per case	Assumption
Willingness to pay, WTP (GDP per capita 2016, Cambodia)	1,270	US$	World Bank
Risk of death for appropriate empirical treatment	0.070		Dataset
Risk of death for inappropriate empirical treatment	0.105		Assumption*
Quality adjusted life year loss	53.5	QALYs	WHO Life table, Cuthbertson et al. 2010

* To our knowledge, there have been no studies estimating the attributable risk of death in children with invasive bacterial infections due to delays in starting appropriate antibiotics. Based on consideration of the adult literature, we make the conservative assumption that the odds ratio for increased mortality due to delay in effective treatment is 1.5.

References

1. Drug Medical Supply and Information Center (DMSIC), Ministry of Public Health Thailand 2016. Available at: http://dmsic.moph.go.th/dmsic/admin/files/userfiles/files/mp2_2559_Announcement_071159.PDF
2. Marquet K, Liesenborgs A, Bergs J, Vleugels A, Claes N. Incidence and outcome of inappropriate in-hospital empiric antibiotics for severe infection: a systematic review and meta-analysis. *Critical Care*. 2015;19(1):63.
3. WHO-CHOICE (Choosing Intervention that are Cost-Effective). 2014. Available at: http://www.who.int/choice/costs/CER_thresholds/en/
4. World Bank, GDP per Capita (Cambodia) 2016 Available at: https://data.worldbank.org/indicator/NY.GDP.PCAP.CD
5. WHO Life table, Cambodia (2016) Available at: http://apps.who.int/gho/data/?theme=main&vid=60270
6. Cuthbertson BH, Roughton S, Jenkinson D, Maclennan G, Vale L. Quality of life in the five years after intensive care: a cohort study. Crit Care. 2010;**14**:R6.
