# Supplementary material for: Using machine learning to guide targeted and locally-tailored empiric antibiotic prescribing in a children's hospital in Cambodia
Source: Wellcome Open Res. 2018 Oct 10;3:131. [Version 1] doi: 10.12688/wellcomeopenres.14847.1 (PMC6352926; doi:10.12688/wellcomeopenres.14847.1)
Supplement: Peer review history

# Peer review: Referee response for version 1

**DOI:** 10.21956/wellcomeopenres.16176.r34568

**Published:** 2019-01-29

**Authors:** Randall C Wetzel, Melissa Aczon

**Affiliations:** 1 VPICU, Children's Hospital Los Angeles, Los Angeles, CA, USA

This is a well written and potentially important paper analyzing the results of a Machine Learning approach to predicting antibiotic resistance in a children’s hospital in Cambodia with multiple models including Random Forests. This study compared performance of several off-the-shelf machine learning methods in classifying bacterial infections.  There were 243 cases, 35 independent variables and 4 clinical (binary) outcomes:  Gram stain; resistance to ampicillin & gentamicin; resistance to ceftriaxone; and resistance to both.  The results are pretty much exactly what you would expect (with random forests and GBMs outperforming other methods).   Overall, the paper is well-written and offers some interesting cost and utility discussions.  The methods section reads like a text book comparison of several ML algorithms.

 We applaud this useful work which is well done and well described.

**Methods**:

1. Recommendation for authors:  for each of the 4 tasks, please provide the class-split (resistant or non-resistant, etc) of your data.
2. It is nice to see the full cost analysis, instead of only sensitivity-specificity.

**Results**:

 Figure 4:  Presumably, this calibration plot corresponds to a single iteration (out of the 1000) of the random forest (RF), and that much like the ROCs in Figure 3, there’d be variance to them.  Please state this explicitly in the text.

 In figure 4 how was the calibration plot generated, was this a GiVitI or was a Hosmer Lemehsow methodology used. Check labels and spelling. 

 Figure 5:  Similar to above comment – that this corresponds to a single iteration of the RF?

 Figure 6:  It’s really nice to see this analysis.  However, similar comment again as before:  Is this from the RF model, and if so, a single iteration?

 In general the figures should be similar with labels and formatting to aid the readers understanding. They take a lot of time to digest.

 Were models with fewer, key observations considered.  In figure sic the importance varies by antibiotic.  Where the predictor importance logical – any comments, for example owning livestock and refrigeration are clearly relevant but were these data prospectively gathered.

**Utility**:   It is really good to see this section; it shows the authors are thinking about the problem holistically.

 Congratulations on this nice comparison of ML techniques and a useful approach to suggesting appropriate antibiotic therapy.

We have read this submission. We believe that we have an appropriate level of expertise to confirm that it is of an acceptable scientific standard.

# Peer review: Referee response for version 1

**DOI:** 10.21956/wellcomeopenres.16176.r34056

**Published:** 2018-10-29

**Authors:** Gwen Knight, Quentin Leclerc

**Affiliations:** 1 Centre for the Mathematical Modelling of Infectious Diseases, Department of Infectious Disease Epidemiology, Faculty of Epidemiology and Population Health, London School of Hygiene & Tropical Medicine, London, UK

This study attempts to evaluate the potential of machine-learning techniques to guide empiric antibiotic prescription in hospitalized children in a resource-limited hospital setting. This is an admirable aim as the impact on patient outcomes and resistance could be great.

 The authors gathered data on the antibiotic resistance profile of bacteria infecting patients, as well as a range of information on the patients themselves which only required the use of a simple questionnaire to be obtained. Different machine learning algorithms were trained and tested on these data, and their predictive capabilities were compared. The study successfully shows the potential of these techniques to identify patients with resistant bacteria, but also to avoid prescribing second-line antibiotics when unnecessary. The conclusions are encouraging regarding the applicability of this currently uncommon approach in a wider setting.

 The manuscript is well written, with clarity in explanations. The authors clearly put a lot of effort into making their methods and results as comprehensive as possible, even for an audience without any previous knowledge of machine learning principles. Although the choice of figures is good, to ensure the reader’s comprehension we believe some minor corrections should be made to these, and a few methodological points should be further clarified (see below). These do not alter the main analysis but are only for clarity in further understanding.

*Minor points*

**

*Abstract*

- Throughout: “AUC” is used three times as an abbreviation instead of “AuROC” which is defined as the correct abbreviation in the text, suggest replacing “AUC” with “AuROC”

*Data*

- In terms of the data collected, 35 independent variables seems like a lot – is this unusual for such a hospital to have such data? How generalisable is it?

 

*Methods*

- Could you please expand on why Gram staining was predicted? The reasoning for attempting to predict antimicrobial resistance is clear, but no justification or discussion is given for the importance of predicting Gram stain.
- In “Methods – Data Analysis”, you mention that “missing data for binary predictors were treated as negative”. Could you please justify this choice, and comment on the potential impact that this has on the results? (i.e. might be overestimating the absence of the predictor, which would lead to incorrectly judging its value as a predictor)
- In “Methods – Training the algorithm”: Are “error estimation” and “testing purposes” the same thing? As in were the data split five ways to give the 80/20 split? the second paragraph feels like it shouldn’t be there, aren’t you actually explaining the k fold validation you did in the third one?

 

*Results *

- What is a “biologically impossible value”?
- It was unclear what random forest test set ROC “did not cross with other test set ROC curves” means and whether true or not. As in, by eye, it seems very close to crossing other curves – can you plot this in the supplementary perhaps?
- Figure 4: We found this figure difficult. Mainly, in terms of what the bins mean on the x axis. Did you calculate for each strain what the probability of resistance was and then compare to the actual probability of resistance? The bins then grouped strains with the same probability?
- Figure 4: the points appear to be misaligned with the x-axis?
- Figure 4: spelling mistake in “ampicillin”
- Figure 4&5: Could the titles match Figure 3 (i.e. “Resistant to ampicillin + gentamicin”, “Resistant to both” …)? especially since “neither” might induce the reader in thinking this is looking at fully susceptible bacteria.
- Figure 5: Should the y axis label not be “predictors”?
- Figure 5: To aid understanding, could you add the interpretation you give in the paragraph describing Figure 5’s results i.e. that 100% means reduction in accuracy 100% of the time as I was unsure of the units for the x axis. Could you also expand on the methodology used to assess the relative importance of the predictors?
- Correction: “but household size was found to BE much more important”
- Figure 6: could you make the y axis the same for Figure A & B to make it clearer that the same cutoff is being used? Also, why is the test set on the left and the train set on the right? Wasn’t the train set used to determine the threshold and then tested on the test set? So it makes more sense for them to be switched around?
- Figure 6: what does the vertical dashed line correspond to? It looks like the point that minimises the cost function?
- Figure 6: you include “false negatives (FN)” in the caption, but this doesn’t actually appear in the figure, perhaps consider redoing it with different values to actually show false negatives? (In reality you would want to avoid having these, but here it would be beneficial for the reader’s comprehension)
- Table 1 is not referred to in the text.
- Table 1: “Inf, infinity” in the caption, but this doesn’t appear anywhere in the table
- Online tool – could you also allow the threshold to be varied in this?
- Online tool – what does the variation in the top two plots show? i.e. when you input a new cost value?
- Using the online tool, our understanding of the last result is that the cost function is minimised at a threshold of 0.21 when the WTP to avoid carbapenem use is linked to $200? However, if this WTP decreases the minimum cost also decreases. i.e. the minimum point when, for example WTP is zero, is $2290 but at $200, more like $2350. Could you provide further explanation of this?

 

*Discussion*

- Wider implications: it is unclear to us how machine learning would be needed with rapid diagnostic tests if the latter tell you bug and resistance. Could you expand more on what you think machine learning would add?
- Correction: “potentially grave potential consequence” (remove one)

 

*S3 appendix*

- Incorrectly labelled S4 on the pdf.
- *Table S2*: The “WTP for *avoiding unnecessary* imipenem use” is confusing terminology. At first read, it seems that this should multiply both the TN and the FN. Could you instead somehow link it to the more intuitive first explanation here that imipenem use is linked to a “cost” for future resistance?
- *Table S3*: could you make it clearer that this “WTP” is for QALY gain and different to the WTP for avoiding unnecessary imipenem use?

We have read this submission. We believe that we have an appropriate level of expertise to confirm that it is of an acceptable scientific standard.
